# Silymarin/Silybin and Chronic Liver Disease: A Marriage of Many Years

**DOI:** 10.3390/molecules22020191

**Published:** 2017-01-24

**Authors:** Alessandro Federico, Marcello Dallio, Carmelina Loguercio

**Affiliations:** Department of Clinical and Experimental Medicine, Second University of Naples, 80131 Naples, Italy; marcello.dallio@gmail.com (M.D.); carmelina.loguercio@unina2.it (C.L.)

**Keywords:** silymarin, silybin, antioxidants, alcoholic liver disease, viral hepatitis, non-alcoholic fatty liver disease, hepatocellular carcinoma

## Abstract

Silymarin is the extract of *Silybum marianum*, or milk thistle, and its major active compound is silybin, which has a remarkable biological effect. It is used in different liver disorders, particularly chronic liver diseases, cirrhosis and hepatocellular carcinoma, because of its antioxidant, anti-inflammatory and antifibrotic power. Indeed, the anti-oxidant and anti-inflammatory effect of silymarin is oriented towards the reduction of virus-related liver damages through inflammatory cascade softening and immune system modulation. It also has a direct antiviral effect associated with its intravenous administration in hepatitis C virus infection. With respect to alcohol abuse, silymarin is able to increase cellular vitality and to reduce both lipid peroxidation and cellular necrosis. Furthermore, silymarin/silybin use has important biological effects in non-alcoholic fatty liver disease. These substances antagonize the progression of non-alcoholic fatty liver disease, by intervening in various therapeutic targets: oxidative stress, insulin resistance, liver fat accumulation and mitochondrial dysfunction. Silymarin is also used in liver cirrhosis and hepatocellular carcinoma that represent common end stages of different hepatopathies by modulating different molecular patterns. Therefore, the aim of this review is to examine scientific studies concerning the effects derived from silymarin/silybin use in chronic liver diseases, cirrhosis and hepatocellular carcinoma.

## 1. Introduction

The use of medicines derived from herbal products is an ancient practice in scientific research. There are a lot of molecules able to generate healthy benefits, if used in a large range of diseases. Among these molecules, over the centuries, silymarin has had a very important role [1]. The cultivation of *Silybum marianum* as a plant with potentially healthy effects dates back to the Ancient Egyptian age. Indeed, the discovery of archeological evidence representing *Silybum marianum*, located in the Egyptian Museum, is well known. The remarkable attention to this plant by Egyptians was likely related to its probable role in human health, therefore represented on everyday objects. Another historical reference was found in the Bible, in which the plant is also called “*Lebanon cardus*”. A better description of its characteristics dates back to the Pliny the Elder age (23–79 A.D.): its juice and seeds were used in case of poisoning due to snake bite and in melancholic depression, a pathology that was supposed to be “liver complaint”. After the Middle Ages, the use of *Silybum marianum* in medicine has increased even more, and, at the same time, scientific data derived from its use have intensified, especially in liver pathologies [2].

Silymarin is the extract of *Silybum marianum*, or milk thistle, and consists of seven flavonoglignans (silibinin, isosilibinin, silychristin, isosilychristin and silydianin) and a flavonoid (taxifolin) [3]. Among these substances, silybin is mainly prevalent and has the most important biological effect. It makes up about 70% of the total composition of silymarin in the form of two diastereoisomeric compounds: silybin A and silybin B [4,5].

With respect to pharmacokinetics, silymarin is a low bioavailability compound if administered per os, with a lack in solubility in water. This is due to both its inefficient absorption in the intestine and an elevated metabolism of the first liver passage after its absorption; two mechanisms that decrease haematic concentration and consequently the arrival at the target organ [6,7,8]. However, this limitation has been efficaciously surpassed by the introduction of complexing with phosphatidylcholine that has a better absorption, and new silibinin glyco-conjugates (gluco, manno, galacto, and lacto-conjugates), which have both a high solubility in water and a strong antioxidant power [9,10]. The elevated absorption of these compounds has led to assessing the safety of silymarin in its therapeutic use. Its high tolerability was demonstrated by toxicity studies on animals treated with silymarin for a long time, whereas other studies on humans highlighted, among the most common side effects, its prolonged and high dosage use, headaches and itching [11,12]. No deaths or life-threatening adverse events have been reported [12]. Even if silymarin is a well-tolerated molecule, it is necessary to point out the few cases of scientific evidence in literature that demonstrate potentially harmful effects: in a phase I clinical trial, the use of 13 g per day of silybin in patients affected by prostate cancer was correlated to hyperbilirubinemia and alanine aminotransferase (ALT) increase. Moreover, it should be taken into consideration the possible side effects derived from the influence both on estrogen signaling, a potentially usable function even for therapeutic purposes, and on the aryl hydrocarbon receptor [13,14].

Scientific evidence, achieved so far, allows us to understand the mechanisms of action through which silybin carries out its activity by interacting with various tissues. In this regard, the action of silybin manifests in the modulation of inflammation and apoptosis, which, together with its antioxidant power, represent the key points that led to using it in different pathologies [15,16,17]. Silybin acts through the turning-off of pro-inflammatory signals, derived from nuclear factor-κB (NF-κB) activation, involved in the induction of the synthesis of cytokines such as tumor necrosis factor-α (TNF-α), interleukin (IL)-1, IL-6, and granulocyte-macrophage colony stimulating factor (GM-CSF) [15,16]. Furthermore, silybin induces apoptosis through the modulation of cytoplasmatic levels of bcl-2-like protein 4 (Bax) and B-cell lymphoma 2 (Bcl-2) proteins, cytochrome c release and caspase-3 and 9 activation [17]. The anti-oxidant activity is due to its capacity to act as both free radical scavenging and lipid peroxidation inhibitors, as demonstrated in vitro and in vivo [18,19,20,21,22,23,24].

**Figure 1:**
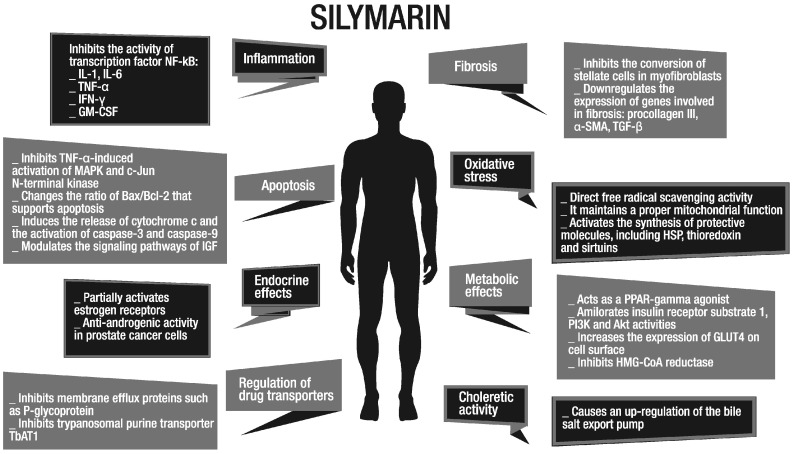
Different therapeutic activities of silymarin. IL-1/6: interleukin1/6; TNF-α: tumor necrosis factor-α; IFN-γ: interferon-γ; GM-CSF: granulocyte-macrophage colony stimulating factor; MAPK: mitogen-activated protein kinase; Bax: bcl-2-like protein 4; Bcl-2: B-cell lymphoma 2; IGF: insuline-like growth factor; α-SMA: α-smooth muscle actin; TGF-β: transforming growth factor-β; HSP: heat shock proteins; PPAR-γ: peroxisome proliferator-activated receptor γ; PI3K: phosphatidylinositol-4,5-bisphosphate 3-kinase; Akt: protein kinase B; HMG-CoA: 3-hydroxy-3-methylglutaryl coenzyme A; GLUT 4: glucose transporter type 4.

Silymarin is also a modulator of estrogen signaling [25], insulin sensitizer [24,26,27,28], regulator of intracellular transport of drugs [29,30,31,32], anticarcinogen [25,33,34,35,36,37,38,39], antidiabetic through signal regulation of peroxisome proliferator-activated receptor γ (PPAR-γ) [40], antifibrotic [41,42,43,44,45] and choleretic [8] (Figure 1).

The great number of actions carried out by silymarin explains the reason why a lot of scientific studies have been performed in order to understand its efficacy in various pathologies [46]. In rheumatic diseases, such as rheumatoid arthritis, silymarin acts as an anti-inflammatory by inhibiting migration and activation of neutrophil in the articulations [47]. In different oncological diseases, such as prostate cancer, cervical cancer, hepatocellular carcinoma (HCC), bladder cancer and lung cancer, silymarin reduces cell vitality and runaway cell replication [48,49,50,51,52]. Because of its detoxifying power, its hydrosoluble endovenous formulation, it is used as an anti-hepatotoxic drug in poisonings due to acetaminophen, arsenic, carbon tetrachloride, butyrophenones, phenothiazines and *Amanita phalloides* toxins [53,54,55,56]. In hypercholesterolemia, silymarin inhibits 3-hydroxy-3-methylglutaryl coenzyme A (HMG-CoA) reductase, reducing cholesterol synthesis [57]. Lastly, in neurological and psychiatric diseases, this molecule acts through the turning-off of inflammatory signals, which underlies the degeneration of dopaminergic neurons in Parkinson’s disease, and it improves the clinical picture ascribable to obsessive-compulsive disorder [58,59]. Of note, the role of herbal products in chronic liver disease, which currently represents one of the most important health problems in about 10% of the world population, is the most studied topic in the scientific community [60]. Indeed, in chronic liver diseases, silymarin acts through different mechanisms and complex biological interactions able to produce benefits in various pathologies, some of which are systemic and can involve the liver. Researchers have studied for a long time the biological effects that natural products such as silymarin have on pathologies such as viral hepatitis, alcoholic liver disease (ALD), metabolic hepatitis, as well as on the common end stages of hepatopathies, that is, cirrhosis and HCC, on which silymarin carries out an important biological action [46,61].

The aim of the present review in literature is to examine the scientific evidence concerning the effects derived from silymarin/silybin use in various etiologies of chronic liver diseases.

We mentioned all papers that evaluate the therapeutic role of silymarin/silybin in chronic liver diseases and pharmacokinetic studies from 1980 to 2016 on these substances (because the studies on this topic belonging to the aforementioned range are the most interesting in the scientific production and pharmacokinetic studies started in 1980), taking into account the methodology used to assess the aim, the route of administration and the composition of silymarin/silybin complexes.

## 2. Silymarin/Silybin in Chronic Liver Disease

### 2.1. Viral Hepatitis

Nowadays, even if a change in the etiology of chronic liver diseases is occurring, different strains of viral hepatitis still represent an important cause of chronic liver damage [62].

The anti-oxidant and anti-inflammatory action of silymarin allows us to understand easily its potentially healthy activity oriented towards the reduction of virus-related liver damage through the softening of inflammatory cascade and immune system modulation [63]. However, the relationship between chronic viral hepatitis and silymarin cannot be limited to this simple approximation. From the analysis of literature, it is possible to deduce the poor quality and lack of studies that analyse the interaction between silymarin and hepatitis B virus (HBV) infection. A meta-analysis performed by Wei et al. evaluated the efficacy and safety of silymarin and its therapeutic combination with antivirals (lamivudine and interferon) in the treatment of HBV chronic hepatitis [64]. The research highlighted that, from the analysed studies, it was possible to deduce a similar efficacy of silymarin and antiviral agents in normalizing aspartate aminotransferase (AST) and ALT levels, as well as an equivalent negative conversion rate of serum HBsAg (Relative Risk (RR) = 1.50; 95% Confidence Interval (CI) = 0.18–12.35) and HBeAg (RR = 1.80; 95% CI = 0.43–7.60). Furthermore, they highlighted that silymarin, associated with the use of antivirals, was able to promote a major effect on serum level reduction of transaminases compared to the use of antivirals alone [64]. Nevertheless, the same authors stated that there was no remarkable data in literature for suggesting the use of silymarin associated with antiviral therapy in the treatment of HBV chronic infection, probably due to various criticism in the construction of analysed trials [64]. Similar outcomes were obtained by other researchers, who highlighted the role of silymarin in inducing a reduction of transaminase levels during viral hepatitis. However, with respect to the histology or serum viral content, there were no direct effects due to its use [47].

Virus C chronic hepatitis (HCV) represents the most frequent cause of viral chronic hepathopathy worldwide, especially after the introduction of HBV vaccination in the 1980s [65].

Although, in clinical practice, most of the patients affected by HCV, who undergo or do not undergo antiviral treatment, use herbal products such as silymarin, its use cannot be recommended because it is not supported by significant scientific evidence [66]. As highlighted by analysis of scientific literature, even for the role of silymarin in determining the block of both entry and fusion HCV and viral replication [67,68,69,70,71,72], in a meta-analysis of Yang et al., a healthy effect on HCV-RNA serum level has been demonstrated (although not statistically significant). This effect was proved only when silymarin was administered both per os and through high-dose intravenous injection [66].

Intravenous administration of silybin is able to inhibit viral replication by intervening directly in the HCV lifecycle. Indeed, it is able to inhibit HCV RNA-dependent RNA polymerase function independently from intracellular interferon (IFN)-induced antiviral pathways [71]. Silymarin is unable to block HCV binding to cells; however, it blocks both HCV entry and fusion of HCV with liposomes [69]. Furthermore, silymarin, but not silybin, inhibits JFH-1 genotype 2a NS5B-dependent RNA polymerase activity, microsomal triglyceride transfer protein activity, apolipoprotein B secretion, and, therefore, the leakage of infectious virion from the cell [69].

The effects on inhibition of viral replication carried out by intravenous administration were also analysed by Ferenci et al., who demonstrated how silybin, by blocking HCV polymerase function at a half maximal inhibitory concentration (IC50) between 75 μM and 100 μM, is able to reduce HCV viral loads from three to four logs within one/four weeks in previous peginterferon nonresponder patients [73].

This fact is confirmed by a case report in which a potential antiviral direct effect carried out by a combined treatment of 238 days with 1200 mg/day of endovenous silymarin, 1200 mg/day of ribavirin and 6000 U/day of vitamin D has been highlighted. This therapeutic approach has been demonstrated to be very tolerable, and it allowed the achievement of sustained virologic response (SVR) in a 44 year-old female HCV genotype-1 infected patient with a previous therapeutic failure based on interferon and ribavirin [74]. Moreover, the endovenous administration of silymarin is able to reduce the viral load of patients affected by genotype 3 HCV, opening the doors to a possible therapeutic combination with the latest direct-acting antivirals (DAA) therapies, in light of the most recent viral eradication data for the different HCV genotypes, towards difficult-to-treat genotypes [75,76,77].

Some years ago, before new therapies based on DAAs, the endovenous treatment with silymarin was also studied as possible adjuvant, lead-in therapy for 14 days (20 mg/kg/day) followed by a triple therapy with peginterferon-ribavirin and telaprevir for 12 weeks, in order to obtain SVR in difficult-to-treat patients: HCV/human immunodeficiency virus (HIV) co-infected patients or with advanced fibrosis, in whom a viral eradication rate of 63% was found, which is higher than deriving data, for the same type of patients, from CUPIC (Compassionate Use of Protease Inhibitors in viral C Cirrhosis) and REALIZE (A Safety and Effectiveness Study of Telaprevir in Chronic, Genotype 1, Hepatitis C Patients That Failed Previous Standard Treatment) (20% and 50%, respectively) studies [78,79,80].

This interesting data has to be re-assessed in light of the latest therapies for the eradication of HCV infection, in which most DDA therapeutic regimens reach rates of SVR higher than 90% and an onset of side effects lesser than previous therapies based on interferon and ribavirin. Consequently, in clinical practice, the implementation of a daily endovenous therapy based on silymarin is not applicable.

### 2.2. Alcoholic Liver Disease

The excessive ethanol consumption represents one of the most widespread causes of chronic hepatopathy worldwide, with a prevalence that varies depending on the geographical area considered. Sometimes, alcohol abuse can associate itself to other causes of liver damage, including HCV, causing a coexistence of harmful stimuli for the liver, which is able to hugely accelerate the progression of the pathology to more advanced forms, as well as cause acute liver failure in patients with HCV-related chronic hepatopathy [81].

Alcohol liver damage is mainly linked to the alteration of the oxidoreductive potential of cells due to ethanol metabolism. Indeed, the activity of alcohol dehydrogenase at first, and of aldehyde dehydrogenase later, causes a reduction in NAD^+^/NADH ratio, which underlies the process that causes a reduced mitochondrial capacity to metabolize lipids. The high quantity of lipids, together with the elevated intracellular oxidative stress, due to activation of secondary metabolic pathways for ethanol, such as microsomal ethanol oxidizing system (MEOS), leads to lipid lypoperoxidation, responsible for the loss of cellular and mitochondrial membrane function, with consequent cellular death [82,83].

The protective effect derived from silybin-phosphatidylcholine complex (SilPho) towards oxidative stress was demonstrated in one of our studies: in vitro, the use of SilPho is able to increase cellular vitality, evaluated by 3-(4,5-dimethylthiazol-2-yl)-2,5-diphenyl tetrazolium bromide assay (MTT), in conditions of oxidative stress induced by the incubation of HepG2 and MKN28 cells, with xanthine oxidase and its substratum called xanthine [23]. Moreover, we highlighted the effect derived from SilPho treatment, which reduced, in conditions of oxidative stress, both lipid peroxidation, evaluated by the measurement of a stress oxidative marker (Malondialdehyde), and cellular necrosis [23].

A study on mice, by Song et al. demonstrated that silymarin (200 mg/kg) was able to reduce oxidative stress, due to the gavage of ethanol 5 g/kg body weight every 12 h for a total of three doses, as well as prevent ALT increase, Glutathione (GSH) decrease, lipid peroxidation and TNF-α increase [84]. However, the lack of a pharmacokinetics assessment of silymarin administered per os represents a limitation in this study, given the low bioavailability of the compound, a condition that generates some doubts on the real role of silymarin regarding the results obtained in the aforementioned study.

Similarly, in another study on mice, it was shown how administration of 250 mg/kg per os of silybin is able to antagonize the increase in thiobarbituric acid reactive substance (TBARS), GSH reduction and the decrease of the content and the activities of superoxide dismutase (SOD), catalase (CAT), glutathione reductase (GR), and glutathione peroxidase (GPx), which are effects linked to ethanol exposure [85].

The key point for the comprehension of pathogenesis of ethanol-induced damage is the triggering of mitochondrial dysfunction caused by both lipid peroxidation and direct toxic effect due to intracellular accumulation of acetaldehyde. Indeed, in mitochondria, the main cellular metabolic reactions occur, many of which are potentially able to produce reactive oxygen species (ROS), especially in conditions of mitochondrial dysfunction, leading to closure of a vicious circle able to cause cellular death [86,87]. The use of silymarin and SilPho is able to optimize mitochondrial metabolic processes and the chain of electronic transport, to increase intracellular SOD activity, and to reduce monoamine oxidase (MAO) activity, definitively leading to the reduction of intracellular ROS levels for the improvement of mitochondrial functionality [88,89,90,91].

Therefore, the essential therapy in hepatopathy caused by ethanol abuse, is the abstinence from alcohol supported by pharmacological therapy, psychological support and counseling. However, from the pathogenic point of view, in the antagonism of alcohol-related liver damage, silymarin could represent a useful support therapy for the improvement of liver metabolic processes en route to breaking drinking habits, but further studies are necessary.

### 2.3. Non-Alcoholic Fatty Liver Disease

Non-alcoholic fatty liver disease (NAFLD) is a potentially evolutive pathology that causes fat accumulation in hepatocytes without other pathological conditions able to generate it, such as viral hepatitis, alcohol consumption, and chronic drug use [92].

With respect to the epidemiology, in the last few years, the incidence of NAFLD has shown an exponential increase in Western countries; on the contrary, a reduction of viral hepatitis has been demonstrated. Therefore, NAFLD will be the most frequent cause of chronic hepatopathy in the near future [92]. Nowadays, NAFLD represents both the second most frequent cause of HCC development and the second most frequent indication for liver transplants [93,94,95,96].

NAFLD pathogenesis involves both genetic and environmental factors, which promote the onset of insulin resistance, that play a key role in metabolic syndrome, a complex systemic condition [97,98,99,100,101,102,103,104].

Currently, the problem to be dealt with concerns the lack of specific therapeutic approaches able to antagonize the progression to severe forms or to intervene by breaking the complex network of pathogenetic events that cause its appearance [105,106]. This problem is even more relevant if we consider the elevated distribution of NAFLD in the pediatric population, in which the elevated life expectancy could lead to progression of the pathology from “simple” fat accumulation into the liver to inflammation, cirrhosis and HCC, which manifests itself in youth compared to HCC observed in viral hepatitis [107,108].

The scientific evidence efficaciously gathered in the latest European Association for the Study of the Liver (EASL)-European Association for the Study of Diabetes (EASD)-European Association for the Study of Obesity (EASO) Clinical Practice Guidelines, highlights an improvement of the histological picture and serum liver enzymes derived from a weight loss of about 7%–10% (**B1** evidence level). The weight loss is obtained by both healthy diet, specifically the Mediterranean diet without consumption of processed food, without food and beverages high in added fructose, and by regulation of macronutrient composition (**B1** evidence level), in addition to an aerobic and resistance exercise (**B2** evidence level) [109,110]. Furthermore, the analysis of potentially usable pharmacological approaches has currently generated controversies and doubts about the real hepatic health effect, as well as about the tolerability due to a long-term use [109].

**Figure 2:**
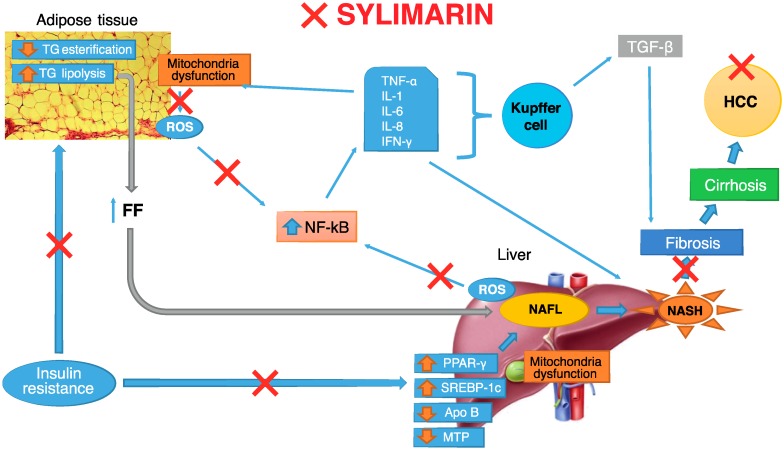
Therapeutic targets of silymarin in non-alcoholic fatty liver disease. TG: triglycerides; ROS: Reactive oxygen species; IL-1/6/8: interleukin-1/6/8; TNF-α: tumor necrosis factor alpha; INF-γ: interferon-gamma; TGF-β: transforming growth factor-beta; NF-κB: nuclear factor kappaB; FF: free fat; NAFL: non-alcoholic fatty liver; NASH: non-alcoholic steatohepatitis; HCC: hepatocellular carcinoma; PPAR-γ: peroxisome proliferator-activated receptor gamma; SREBP: sterol regulatory element-binding proteins; Apo B: apolipoprotein B; MTP: microsomal triglyceride transfer protein.

In this context, different studies have attempted to correlate silymarin/silybin use to the biological effects able to antagonize NAFLD progression, by intervening in various therapeutic targets (Figure 2).

Silybin could be an insulin sensitizer: it is able to reduce intrahepatic fat accumulation, lobular inflammation, ballooning and serum fat, as well as to improve homeostasis model assessment-IR index (HOMA-IR) and insulin tolerance test (ITT) [26]. Moreover, silybin has an important role in reducing visceral fat accumulation, in inducing lipolysis through the transcription of the adipose triglyceride lipase (ATGL) gene and inhibiting gluconeogenesis for silencing of some genes involved in the aforementioned metabolic pathway [26]. Nevertheless, in this work, the timing for the administration of pure silybin is not clear, that is without molecules that increase its oral bioavailability, and high-fat diet (HFD) in the group of rats fed with HFD+silybin. Therefore, taking into account the low bioavailability of pure silybin, if administered per os [111], it cannot exclude that the outcome observed in this study could depend on a reduction of absorption of fat contained in HFD mediated by the formation of non absorbable complexes with silybin, rather than depending on its real role in interrupting the pathogenetic mechanisms that are responsible for NAFLD.

The effect of insulin in determining its biological effects is correlated to the activation of pathway insulin receptor substrate 1 (IRS-1)-phosphatidylinositol-3-kinase (PI3K)-protein kinase B (Akt) able to generate the activation of substrates such as Akt substrate 160 and consequently the expression of glucose transporter type 4 (GLUT4) on the cell surface [112]. The increase of phosphorylation in IRS-1 and PI3K serine, as happens in insulin-resistance, is mediated by the c-Jun N-terminal kinase (JNK) and nuclear factor-κB kinase β (IKK-β) activation, as happens in experimental models of insulin-resistance induced by HFD or palmitic acid [27,113,114]. The treatment with increasing doses of silybin (16, 40 and 100 μg/mL), in vitro, is able to generate an increase in glucose captation, induced by insulin in a model of palmitate-induced insulin-resistance on myoblast C2C12 cells, in which the role of silybin is crucial in the increase of PI3K activity [27].

In a preliminary observation study of our group in 2006, we highlighted how the treatment with silybin-vitamin E-phospholipids complex (376 mg of silybin, 776 mg of phosphatidylcholine, and 360 mg of vitamin E/day) for six months is able to improve ultrasonography of bright liver, liver enzyme levels, HOMA-IR and serum indices of liver fibrosis [28].

The association of silybin with phosphatidylcholine forms a silybin-phytosome that is able to surpass the intestinal wall easily and reach the liver in an elevated quantity. However, the use of a silybin-vitamin E-phospholipids complex does not produce effects on the metabolism velocity of the first liver passage of silybin. Vitamin E is not used for antioxidant purposes, given its low dosage, but it is a chemical stabilizer for phytosomes.

This preliminary result was confirmed in a multicenter, phase III, double-blind clinical trial on 180 patients with histological diagnosis of NAFLD/non alcoholic steatohepatitis (NASH), with a few HCV positive patients (36 patients). Data of 138 patients were analysed, demonstrating that the administration of silybin-vitamin E-phospholipids complex (188 mg of silybin, 388 mg of phosphatidylcholine, and 180 mg of vitamin E/day) for 12 months was able to normalize transaminase levels and improve γ-GT levels and steatosis degree at ultrasonography, although in no statistically significative way. Furthermore, the treatment is correlated to the improvement of glycemia without food, insulinemia and HOMA-IR. In 35 patients treated with silybin-vitamin E-phospholipids complex, a clear improvement of steatosis, lobular inflammation, ballooning and liver fibrosis was demonstrated, compared to baseline, through the execution of a second liver biopsy at the end of the twelfth month period [115].

Patients with NASH and apparently similar clinical/biochemical characteristics could differ in the response to a possible therapy with silybin-vitamin E-phospholipids complex in relation to the level of serum lipid peroxidation (TBARS) markers and NAFLD activity score at baseline. In this way, it is possible to identify the patients with a higher possibility to obtain a benefit from the treatment, also highlighted by serum lipidomic profile analysis [116].

The metabolic activity of silymarin also manifests itself through a moduation of genes involved in lipid metabolism, as well as in the control of oxidative stress. The use of silymarin for four weeks in diet-induced obesity mice showed a clear improvement of plasma levels of triglycerides (TG); low-density lipoproteins (LDL) and high-density lipoproteins (HDL), as well as the improvement of intrahepatic lipid accumulation and NAS score [117]. These results are not ascribable either to a reduced daily calorie intake or to a weight loss, potentially achievable during the treatment.

Therefore, the intervention of silymarin in NAFLD clearly manifests itself through its action on different therapeutic targets involved in the pathogenetic process that leads to its onset and worsening. In this regard, silymarin is probably one of the most promising molecules, usable in NAFLD therapy, that acts on multiple therapeutic targets and has a high safety profile, given the necessity of a long-term treatment. However, the use of silymarin in daily clinical practice could benefit from scientific evidence acquired by trials based on populations of both NAFLD patients and pediatric ones.

### 2.4. Cirrhosis and Hepatocellular Carcinoma

Liver cirrhosis and HCC represent common end stages of different hepatopathies. Firstly, inflammation plays an important role in the induction and worsening of fibrosis. The activation of transcription factors, such as NF-κB, and then the synthesis of proinflammatory cytokines, a condition that underlies all types of chronic liver damage, induces an increase in the transcription of genes involved in the deposition of extracellular matrix proteins from stellate cells, and of TGF-β from Kupffer cells. This deposition, along with the increased synthesis of the tissue inhibitor of metalloproteinases (TIMPs) subverts the structure and function of the liver, leading to the pathological picture of cirrhosis [118]. Even insulin resistance, typical in NAFLD, could have a potential profibrotic effect. Indeed, JNK activation, a very frequent condition in insulin resistance, is able to induce the secretion of inflammatory cytokines and activation of stellate hepatic cells [113,114,119].

Silymarin effectively interferes with the fibrogenetic process: its administration for four weeks, in Wistar rats, at doses conventionally used for therapeutic purposes in hypertransaminasemia in daily clinical practice (50 mg/kg for mice) would be able, in a model of CCl4-induced liver fibrosis, to reduce hepatocyte damage, oxidative stress markers, fibrosis score and tissue hyaluronic acid and the activation of both HSC and Kupffer cells [120]. The main reason linked to the aforementioned observation, should be found in the cytoprotective effect, as well as the antioxidant one, which is carried out by silymarin. Indeed, the reduction of cell necrosis, commonly associated with chronic inflammatory conditions, causes a minor release of factors able to activate HSC, including TGF-β, TNF-α, IL-1, IL-6, ROS, etc., and breaks the vicious circle that increases the inflammation itself.

A crucial role in the progression of liver damage to the development of fibrosis and cirrhosis is carried out by platelet-activating factor (PAF), whose production, strictly related to the coexistence of tissue inflammation, allows HSC to produce a large quantity of collagen [121,122,123,124]. The capacity of remodelling ascribed to PAF, would be opposed by the process of acetylation supported by lysophosphatidylcholine acyltransferase enzymes (LPCAT), whose expression is clearly lower in cirrhotic patients compared to controls. In this regard, silybin is able to antagonize the profibrotic effect carried out by PAF through the increase of LPCAT expression, as well as through a direct reduction of PAF in cirrhotic Wistar rats [125].

The alteration of lipid metabolism due to the mitochondria in conditions of mitochondrial dysfunction, could represent one of the mechanisms that allows the progression of different types of chronic liver damage, such as NAFLD and secondary biliary cirrhosis [126,127]. Mitochondrial dysfunction itself, associated with some liver pathologies, is able to induce an increase in ROS production, leading to a worsening of cell damage. In this regard, silybin promotes mitochondrial functionality of cirrhotic livers by inducing an improvement of mitochondrial citrate transport, and, consequently, by promoting ROS elimination and the efficiency of mitochondrial transport chain [128].

In our in vivo study, the treatment with silybin-vitamin E-phospholipids complex for 12 weeks was able to reduce liver fibrosis in 35 patients who underwent liver biopsies at baseline, and, at the end of the treatment, generating data that has not yet been scientifically assessed in literature by other research groups [115].

The effect of silymarin in antagonizing the deposition of fibrotic tissue must be exclusively considered with regard to de novo fibrogenesis in conditions of chronic liver damage. An advanced fibrosis is an irreversible condition. Consequently, it is not possible to treat it with drugs [129]. In these types of patients, the use of silymarin does not produce any healthy effects. Therefore, the administration of silymarin for antifibrotic effects in chronic hepatopathies could improve the clinical picture only if it is carried out in an early stage of the pathology.

Liver cirrhosis, independently from the etiology of chronic liver damage, is a risk factor for HCC development. The incidence of HCC has increased in the last years in developed countries of the world, becoming the second cause of death due to neoplastic diseases worldwide, since it is responsible for about 750,000 deaths per year [130]. The pharmacological approach in HCC is currently represented by therapy with Sorafenib, a receptor tyrosine kinase inhibitor, used in advanced and metastatic forms of the pathology. The increased lifespan obtained by this therapy, is variable depending on the analysed data and the patient performance status, reducing, however, the quality of life due to the side effects related to the therapy [131].

In an *N*-nitrosodiethylamine (NDEA)-induced rat model of HCC, Gopalakrishnan et al. demonstrated that the treatment with silymarin was able to modulate efficaciously different molecular patterns in an anticarcinogenic sense [132]. Rabbit anti-proliferating cell nuclear antigen (PCNA) polyclonal antibody at a dilution of 1:50 is a cell-cycle marker: its elevated expression is commonly associated to cell hyperproliferation [133]; similarly, argyrophilic nucleolar organiser region (AgNOR) levels are directly correlated to the passage from G1 to S phase of the cell cycle [134]. Silymarin is able to reduce not only PCNA and AgNOR levels compared to controls, but also serum and liver glycoproteins/glycoconjugates content, which could be increased in many neoplastic diseases [132,135,136,137,138]. Silymarin also reduces levels of β-catenin, which, moving into the nucleus, promotes the expression of pro-proliferative genes [139], to reduce Bcl2/Bax ratio, promoting apoptosis, and reduces mitochondrial membrane potential probably associated to cytocrome c release in cytoplasm [132].

Lah et al. proved that silybin significantly reduces HuH7, HepG2, Hep3B, and PLC/PRF/5 human hepatoma cells growth by increasing cyclin-dependent kinase inhibitor p21 and p27/cyclin-dependent kinase (CDK) 4 complexes, by reducing retinoblastoma protein (Rb)-phosphorylation and transcription factor E2F1/transcription factor dimerization Partner (DP) 1 complex, as well as by promoting induction of codifying genes for caspase 3–9 and reducing the levels of survivin, whose sovraexpression is associated with a reduction of cell death [140,141].

Moreover, the antineoplastic effect of silybin could be correlated with increased activity of phosphatase and tensin homolog deleted on chromosome ten (PTEN) and decreased p-Akt production with a modulation of signaling of extracellular signal-regulated protein kinases 1 and 2 (ERK1/2), and, finally, with an anti-angiogenetic effect, as highlighted by the reduction of the expression of the cluster of differentiation (CD)-34 and matrix metalloproteinase (MMP)-2, which represent the markers of angiogenesis and metastatic invasion [141,142,143,144]. 

The analysis of literature shows a large quantity of preclinical in vitro and in vivo studies, which efficaciously prove the potential molecular target on which silybin acts for anticarcinogenic purposes. Indeed, silybin could interfere with the process of tumoral induction, through inflammatory cascade regulation and by decreasing ROS genotoxic potential. Moreover, it could also interfere with tumoral promotion by blocking most of the signaling pathways activated in HCC. Finally, silybin is able to improve the quality of life of patients who have undergone conventional treatment with Sorafenib in advanced forms of HCC.

Nevertheless, there is a strong limitation regarding its large-scale therapeutic use that derives from both the lack of data obtained in trials on a large population of patients and the difficulty conforming the animal model, used in most of the studies, to the human one.

## 3. Conclusions

The “marriage of many years” that links silymarin/silybin to liver diseases, derives from the progressive evidence that, with the passing of time, has led to investigation of, firstly empirically and then scientifically, the mechanisms through which they act in carrying out the therapeutic effect. The studies of pharmacokinetics and pharmacodynamics on silymarin have improved, in the last few years, its applicability in different pathologies, especially liver diseases, allowing, through the use of conjugates compounds, a more efficient application. Through the analysis of literature, it has been demonstrated that silymarin has an effect that allows its use in all of the most frequent causes of liver damage. Indeed, silymarin has three important activities: anti-inflammatory, antioxidant and pro-apoptotic, which represent the “functional triad” that allows for antagonizing the onset and the progression of mechanisms of damage that are responsible for the progression of hepatitis to cirrhosis and HCC. However, it is clear that, also in the end stages of liver pathologies, silymarin can act by limiting de-novo fibrogenesis and antagonizing procarcinogenic mechanisms that cause HCC. Nevertheless, the treatment with silymarin/silybin in routine clinical practice is strongly limited, since it is necessary to obtain scientific data deriving from well-structured trials based on large populations of patients, and to achieve a standardization of methods used for evaluating the therapeutic efficacy, especially in an NAFLD context, that is particularly promising.
